# Plastome data analysis of Cucumis melo subsp.agrestis

**DOI:** 10.6026/97320630017646

**Published:** 2021-06-30

**Authors:** Mohammad Ajmal Ali, Khalid Mashay Al-Anazi, Waquar Akhtar Ansari, Joongku Lee

**Affiliations:** 1 Department of Botany and Microbiology, College of Science, King Saud University, Riyadh-11451, Saudi Arabia; 2 Genetics Laboratory,Department of Zoology, College of Science, King Saud University, Riyadh-11451, Saudi Arabia; 3 Department of Botany, Banaras Hindu University, Varanasi-221005, India; 4 Department of Environment and Forest Resources, Chungnam National University, Daejeon 34134,Republic of Korea

**Keywords:** Cucumis melo ssp. agrestis, Cucurbitaceae, melon, plastome

## Abstract

It is of interest to refine the taxonomic status of C. melo ssp. agrestis using its plastome data. The chloroplast size and GC% was found to be 1,56,016 bp and 36.92% respectively in Cucumis melo subsp. agrestis. The plastome of C. melo subsp. agrestis comprises of two inverted repeat (IR) regions of 25,797 bp each. It consisted of 133 genes with 88 protein-coding genes, 8 rRNA genes and 37 tRNA genes. Analysis of the C. melo ssp. agrestis plastome data will help breeders to improve the yield the crop.

## Background

The cucurbits are vegetable crops of the Cucurbitaceae family. This family carries total 98 genera and 1000 species. Muskmelon (Cucumis melo L.) is a member of the family Cucurbitaceae. The genus Cucumis possess large phenotypic diversity with C. hystrix, C. callosus, and C. sativus var. hardwickii [1]. A number of cucurbits including Cucumis melo subsp. agrestis are used in the indigenous system of medicine. Cucurbitacins in cucurbits possess renowned biological attributes [2]. Information on the reproductive biology of Cucumis melo subsp. agrestis is known [14]. Therefore, it is of interest to refine the taxonomic status of C.melo ssp. agrestis using next generation sequencing (NGS) plastome data for further application [3].

## Methodology

### Plant material, DNA extraction, sequencing, assembly and annotation:

**Figure 1:**
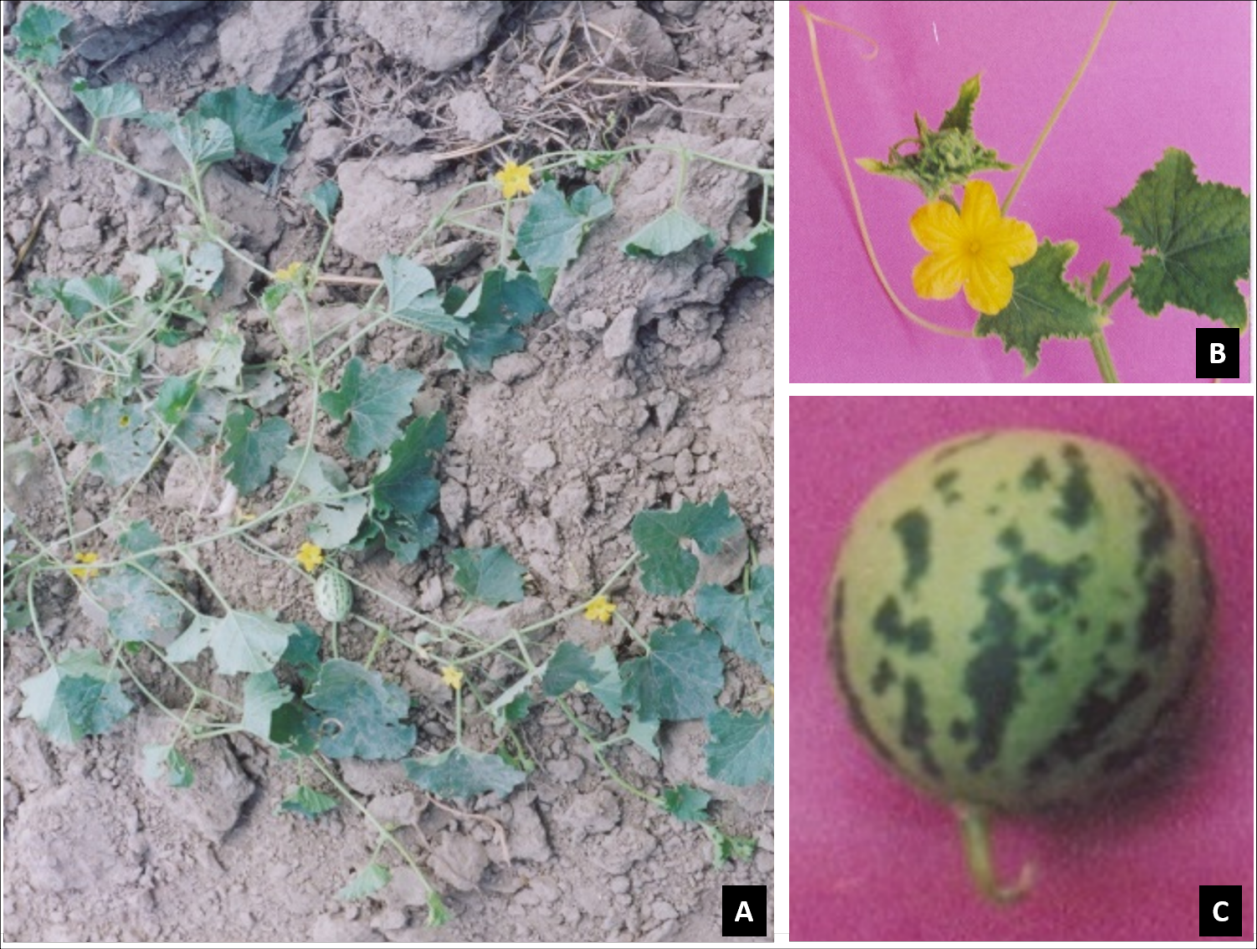
Cucumis melo subsp. agrestis. A) Habit in reproductive stage; B) Flowering; C) Fruit

**Figure 2:**
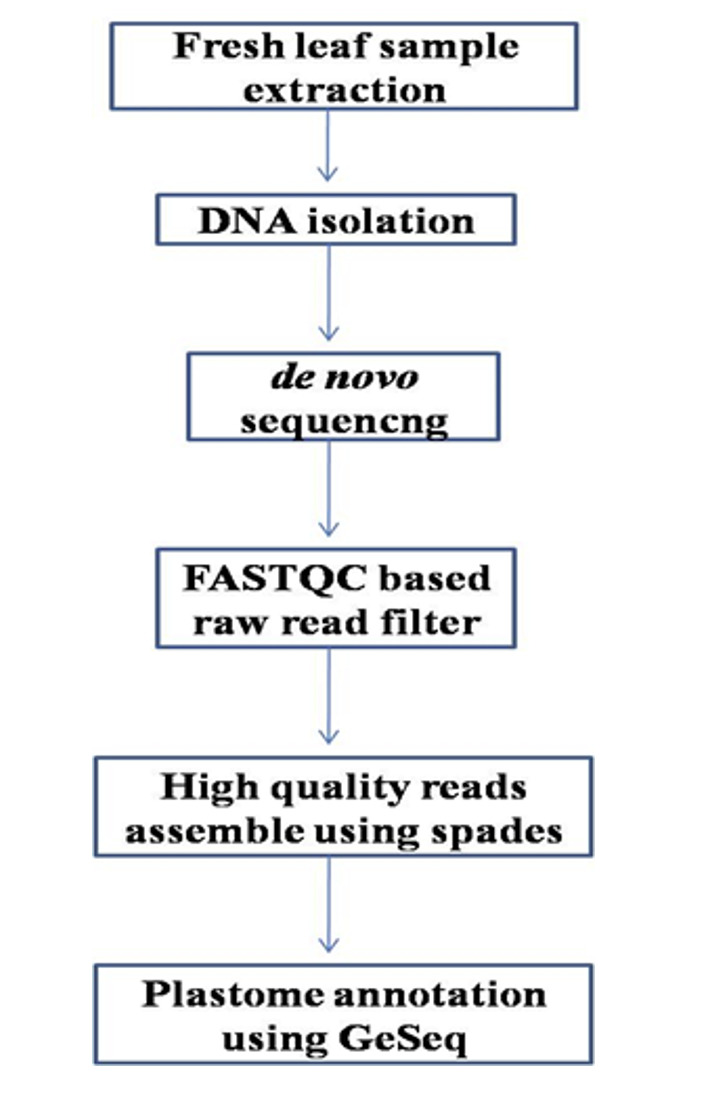
Methodology flowchart

The fresh leaves sample of Cucumis melo subsp. agrestis (Figure 1) was collected from Nita Akaha, Bhagalpur, Bihar India, and fixed in 60-120 mesh size powder silica gel. The DNA extraction was performed using DNeasy Plant Mini Kit (QIAGEN) as per protocol. The de novo sequencing as a single end run of 51 bp was performed (# Illumina platform) at Macrogen, Republic of Korea. The Illumina Pipeline 1.3.2 was used. The FASTQC was used to filter the raw reads. The filtered high quality reads were assembled using SPAdes [4]. The assembled plastome data was annotated using GeSeq [5] as shown in Figure 2.

### Comparative analyses:

The plastome of a total number of 49 species of the family Cucurbitaceae was downloaded from NCBI (Table 1 - see PDF). The variation of plastome was analyzed. The percentage of GC and CDS number were plotted. The plastome of C. melo subsp. agrestis and C. melo were aligned with reference C. sativus using BRIG [6].

## Results and Discussion:

**Figure 3:**
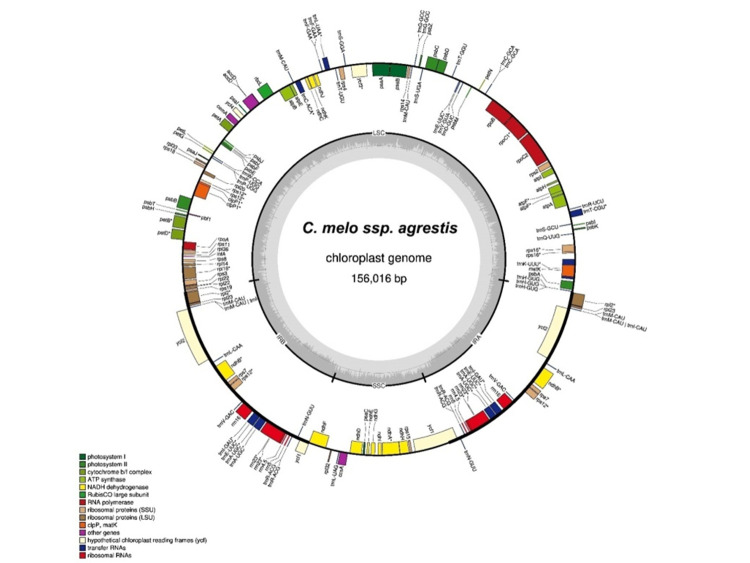
The plastome genome map of Cucumis melo subsp. agrestis.

**Figure 4:**
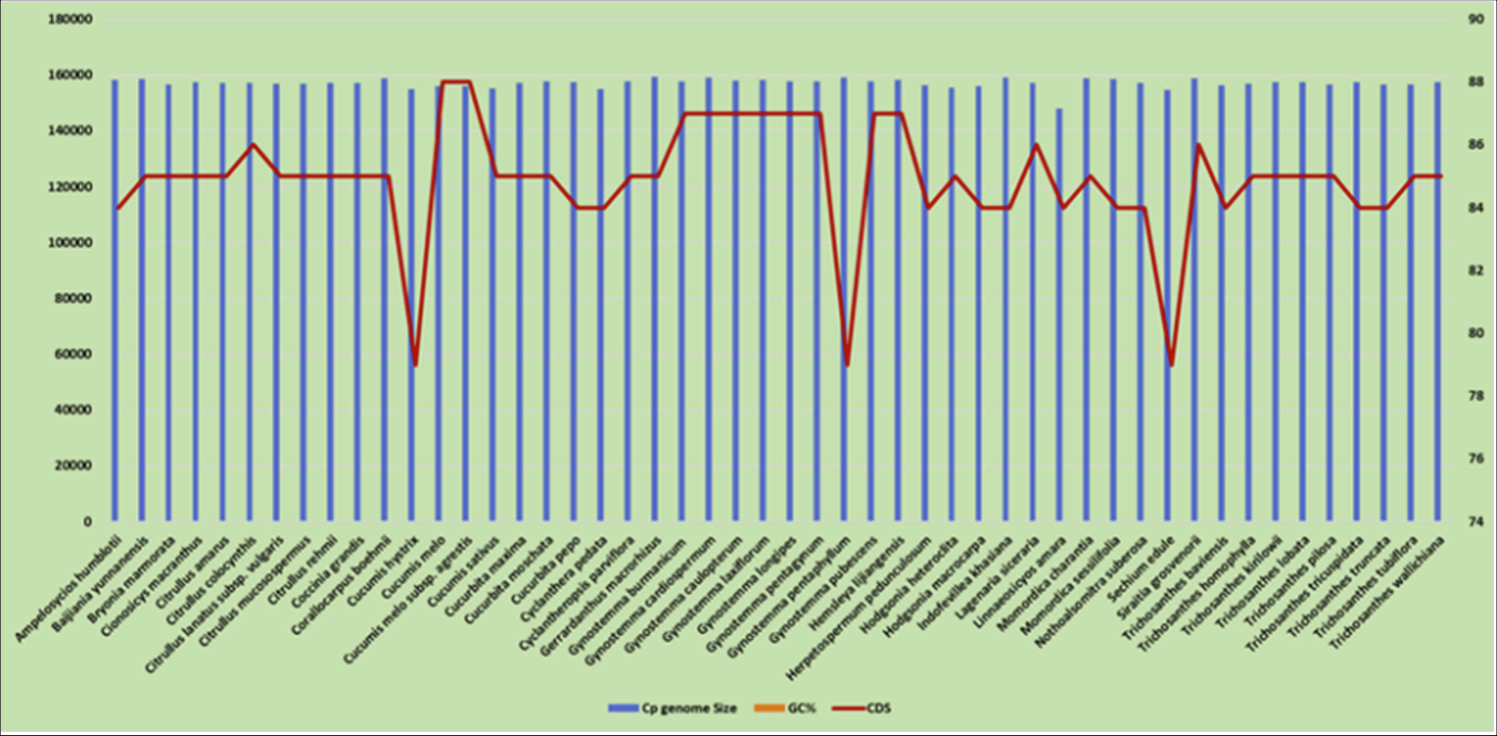
Pattern of cp genome size, GC% and number of CDS across the cucurbits

**Figure 5:**
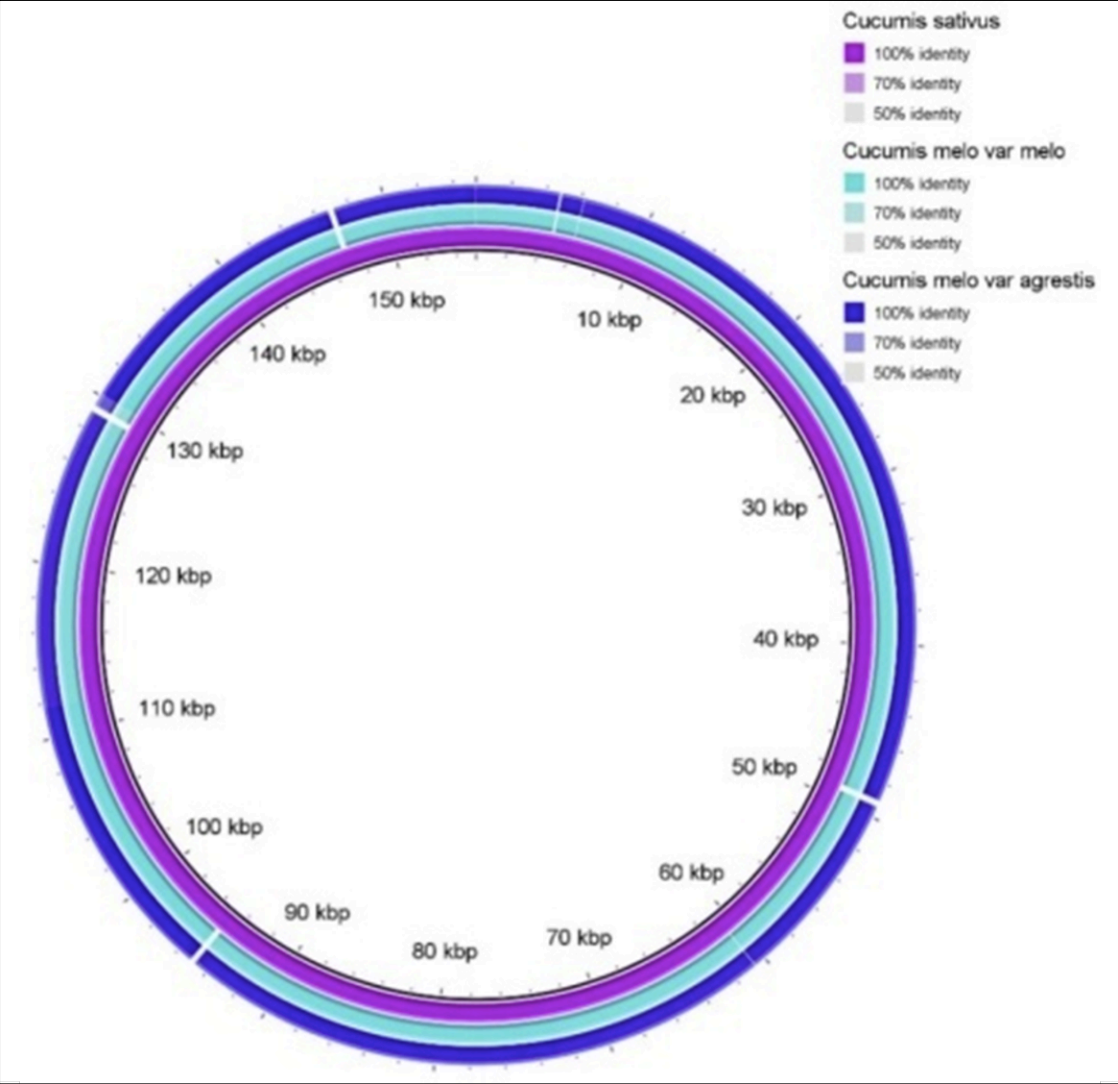
The plastome of Cucumis melo subsp. agrestis and Cucumis melo aligned with reference C. sativus.

The plastome of C. melo subsp. agrestis comprise (Figure 3) of 1,56,016 bp circular DNA carrying 25,797 bp each of two inverted repeat (IR) regions, divided by large single-copy (LSC) regions of 86,335 bp and small single-copy (SSC) regions of 18,088 bp, respectively. It contained a GC% of 36.92, a total number of 133 genes, including 88 protein-coding genes, 37 tRNA genes, and eight rRNA genes. This data is consistent with Cucumis hystrix (size 1,55,031 bp, GC% 36.98%, and 79 CDS), Cucumis melo (size 1,56,017 bp, GC% 36.92%, and 88 CDS), Cucumis sativus (size 1,55,293 bp,GC% 37.07%, and 85 CDS). The plastome size varies from 1,59,232 bp in Gerrardanthus macrorhizus to 1,47,874 bp in Linnaeosicyos amara, the differences of GC% was recorded in the range of 36 to 37%, Ampelosycios humblotii was recorded with minimum 79 CDS,while maximum 88 CDS was recorded in Trichosanthes wallichiana (Figure 4). The plastome of C. melo subsp. agrestis and C. melo aligned at reference C. sativus revealed 100% similarity (Figure 5). The agrestis group melons are monoecious; fruits, typically lightgreen, round, elliptic or oval, smooth surface with dark-greens spots and with numerous small seeds. The phylogenetic analyses suggested the C. melo comprise of various cultivar-groups [1]. The genus Cucumis comprise of many economically significant crops, for example cucumber and melon with several important landraces [7]. The phylogenetic analyses of plastome data of Cucumis species also show proximity of C. melo subsp. agrestis with C. melo [8]. Variation at phenotypic level between wild and cultivated species is evident e.g. tomato [9], sunflower [10], rice [11], Cucumis [12- 13],pepper [9], wheat [10], maize [10], attributed to genetic loci. The comparative transcriptomics showed the similarity at genetic level between the cultivated cucumber (C. sativus) and its wild relative (C. hystrix), the changes in transcription levels which may include alteration in stress tolerance to different abiotic stresses including salinity, heat, cold [7] resistance are continuous during the domestication process [9-11].

## Conclusion

We document the plastome data analysis of Cucumis melo subsp. agrestis to glean insights on crop breeding.
